# Transcriptional Regulation by CpG Sites Methylation in the Core Promoter Region of the Bovine *SIX1* Gene: Roles of Histone H4 and E2F2

**DOI:** 10.3390/ijms19010213

**Published:** 2018-01-16

**Authors:** Dawei Wei, Anning Li, Chunping Zhao, Hongbao Wang, Chugang Mei, Rajwali Khan, Linsen Zan

**Affiliations:** 1 College of Animal Science and Technology, Northwest A&F University, Yangling 712100, Shaanxi, China; weidawei@nwafu.edu.cn (D.W.); lianning@nwafu.edu.cn (A.L.); zhao.chunping@nwafu.edu.cn (C.Z.); wanghongbao@nwafu.edu.cn (H.W.); meichugang@nwafu.edu.cn (G.M.); rajwalikhan@nwafu.edu.cn (R.K.); 2 National Beef Cattle Improvement Center, Northwest A&F University, Yangling 712100, Shaanxi, China

**Keywords:** *SIX1* gene, promoter, DNA methylation, histone H4, E2F2

## Abstract

DNA methylation is a major epigenetic modification of the genome and has an essential role in muscle development. The *SIX1* gene is thought to play a principal role in mediating skeletal muscle development. In the present study, we determined that bovine *SIX1* expression levels were significantly higher in the fetal bovine group (FB) and in undifferentiated Qinchuan cattle muscle cells (QCMCs) than in the adult bovine group (AB) and in differentiated QCMCs. Moreover, a bisulfite sequencing polymerase chain reaction (BSP) analysis of DNA methylation levels showed that three CpG sites in the core promoter region (−216/−28) of the bovine *SIX1* gene exhibited significantly higher DNA methylation levels in the AB and differentiated QCMCs groups. In addition, we found that DNA methylation of *SIX1* core promoter *in vitro* obviously influences the promoter activities. An electrophoretic mobility shift assay (EMSA) and chromatin immunoprecipitation (ChIP) assay, in combination with site-directed mutation and siRNA interference, demonstrated that histone H4 and E2F2 bind to the −216/−28 region and play important roles in *SIX1* methylation regulation during development. The results of this study provide the foundation for a better understanding of the regulation of bovine *SIX1* expression via methylation and muscle developmental in beef cattle.

## 1. Introduction

DNA methylation and histone modification play important roles in gene expression and reprogramming of muscle development. In mammals, methylation of CpG islands represses the initiation of transcription in somatic cells [1,2]. CpG sites are asymmetrically distributed into CpG islands, which are often located in the promoter regions and occur in roughly half of all protein-coding genes [3]. Only a small region within CpG islands in the promoter regions is normally methylated [4] and associated with gene silencing by transcriptional regulation [5]. More recent genome-wide studies have confirmed that transcription factor binding can be strongly influenced by methylation of CpG sites within their recognition sequences [6].

The *SIX1* homeobox gene belongs to the SIX homeodomain family of transcription factor (TF) and is involved in controlling the development of multiple tissues [7,8,9]. More importantly, the function of the *SIX1* gene is tied to skeletal muscle development and regeneration [10,11]. Previous studies have shown that *SIX1* participates in myogenesis during muscle regeneration in coordination with myogenic regulatory factors (MRFs) [11,12]. *SIX1*-null mice die at birth due to hypoplasia and an abnormal structure during primary myogenesis caused by the delayed activation of *MyoD* and a reduction in myogenin genes expression in the limb buds [11,12]. In addition, *SIX1* can be activated by the phosphorylation of its cofactor (Eya), which drives the transformation of the slow-twitch muscle phenotype towards the fast-twitch (glycolytic) phenotype [7]. In a previous study, we demonstrated that the *SIX1* gene was significantly and positively correlated with body measurement traits in Qinchuan cattle [13]. Furthermore, *SIX1* is regulated by MyoD, PAX7, CREB and myogenin TFs and is closely related to DNA methylation during myoblast differentiation [14,15].

Despite the clear role of *SIX1* in transcriptional regulation and the functional formation of muscles and other tissues, there is limited information regarding the influence of CpG methylation on transcriptional regulation of bovine *SIX1* during development and myogenesis. The objectives of this study were to determine the methylation levels of the bovine *SIX1* gene and to confirm potential cis-acting elements with respect to methylation regulation during the developmental stages of Qinchuan cattle.

## 2. Results

### 2.1. Expression Pattern of Bovine SIX1

**Figure 1:**
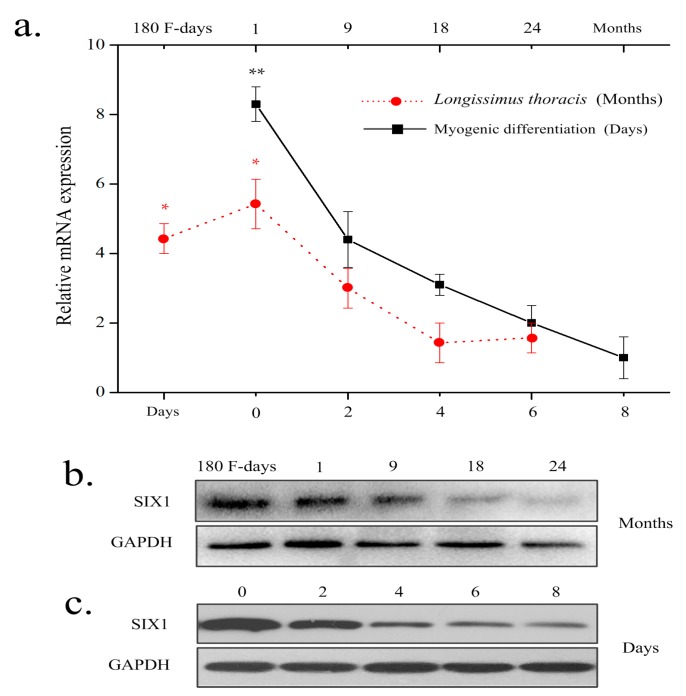
Expression pattern analysis of bovine *SIX1*. (**a**) *SIX1* mRNA expression was determined at different developmental stages. The samples of the longissimus thoracis were obtained at 180 days in utero and at 1, 9, 18, and 24 months after birth (red line) and the bovine myoblasts after the induction of myogenic differentiation at Day 0 (D0), Day 2 (D2), Day 4 (D4), Day 6 (D6), and Day 8 (D8) (black line). *GAPDH* was used as a housekeeping gene; (**b**,**c**) Protein expression pattern of bovine SIX1 was detected in different stages of development and myogenesis. The results are expressed as the means ± SD. “*” *p <* 0.05 and “**” *p <* 0.01.

To assess the expression profile of the bovine *SIX1* gene, mRNA and protein were collected from longissimus thoracis muscle at different developmentalstages (at 180 days in utero and at 1, 9, 18, and 24 months after birth) and the Qinchuan cattle muscle cells (QCMCs) indifferent differentiation stages (0, 2, 4, 6, and 8 days). The result showed that *SIX1* expression in the longissimus thoracis at 180 days in utero and at 1 month of age was significantly higher than during any other stage. In addition, *SIX1* expression was subsequently down regulated by the time myoblasts formed myotubes, while the highest expression occurred during the undifferentiated stage (Figure 1a–c). These results indicate that *SIX1* is highly correlated with individual muscle development of Qinchuan cattle. The primers used in this study are listed in Table 1.

### 2.2. DNA Methylation Analysis by Bisulfite Sequencing Polymerase Chain Reaction (BSP)

**Figure 2:**
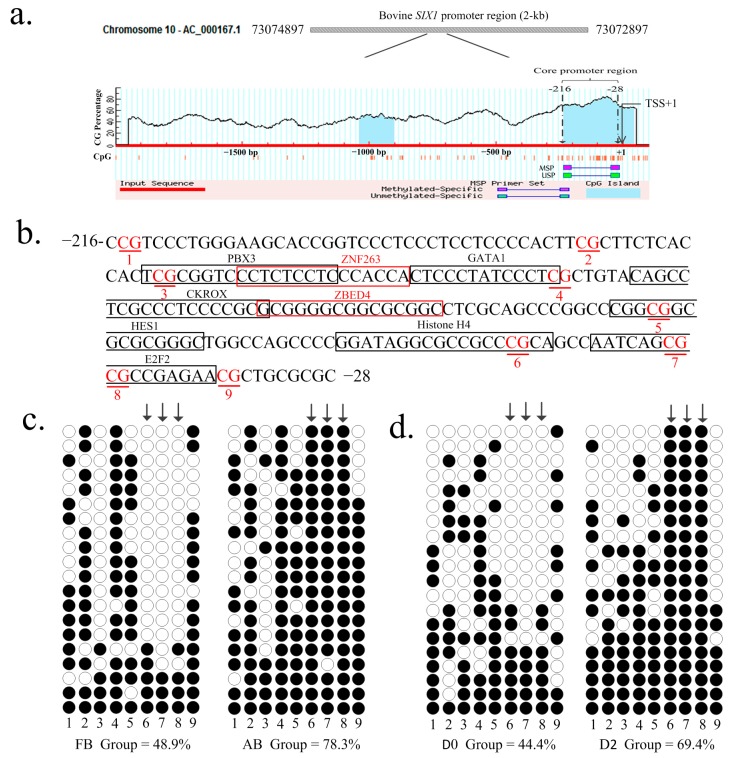
Methylation analysis of the bovine *SIX1* gene in different developmental stages of individuals and cells. (**a**) Schematic representation of the *SIX1* promoter. The bovine *SIX1* gene is located on chromosome 10 and a 2-kb promoter region span from 73072897 to 73074897 (NCBI accession AC_000167.1). Blue background indicates the GC percentage and dashed lines show the core promoter region.The *x*-axis denotes the bp position in the 5′ untranslated region relative to transcriptional start sites (TSS). MSP and USP denote the methylated-specific primer and unmethylated-specific primer, respectively. (**b**) The sequence of the core promoter of the bovine *SIX1* gene. The methylation loci are marked in red letters and putative transcription factor binding sites are boxed. (**c**,**d**) The percentages of the 9 CpG sites at different stages were analyzed using QUMA software. FB denotes the fetal bovine group and AB denotes the adult bovine group; D0 and D2 denotes QCMCs inducing stages. Each line represents one individual bacterial clone, and each circle one single CpG dinucleotide. Open circles and black circles show unmethylated and methylated CpGs, respectively.

Using online software MethPrimer (http://www.urogene.org/methprimer/), a CpG island was predicted to be located in the core promoter (−216/−28) of the *SIX1* gene (Figure 2a), which had been identified in our previous study. Methylation patterns of the CpG sites at core promoter of *SIX1* were determined using bisulfite-assisted sequencing at different stages of for individual animals or in QCMCs. In total, 9 CpG sites (Figure 2b) were identified in all clones. The methylation percentages of the 9 CpG sites were determined in two key stages of myogenesis (myoblast differentiation at Day 0 and Day 2) and muscle maturation (at 180 days in utero and at 24 months after birth) and assessed using QUMA software (http://quma.cdb.riken.jp/). As shown in Figure 2c,d, the DNA methylation levels changed a great deal between the FB and AB animals (resulting in 48.9 ± 4.94% and 78.3 ± 5.36%) and in undifferentiated and differentiated stages of QCMCs (resulting in 44.4 ± 5.88% and 69.4 ± 6.55%). However, methylation percentages of the 9 CpG sites did not significantly change between other stages of myogenesis (D2, D4, D6, and D8) or muscle maturation (1, 9, 18, and 24 months). Thus, the present study mainly focused on the groups of myoblast differentiation at Day 0 and Day 2, at 180 days in utero, and at 24 months after birth.

**Figure 3:**
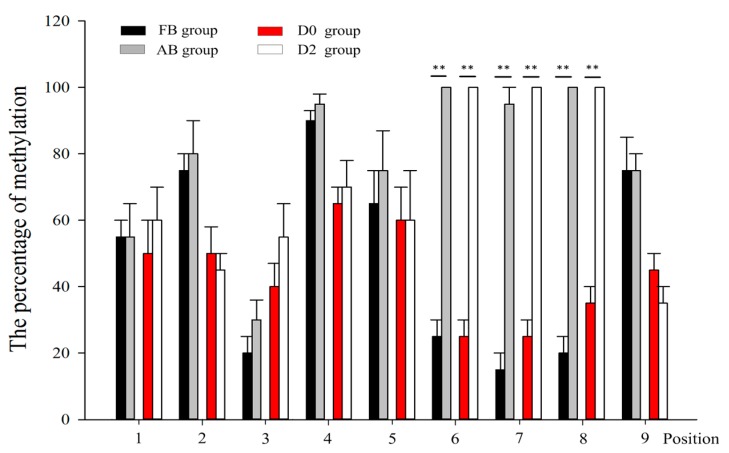
Methylation levels of the *SIX1* gene core region containing 9 CpG sites were analyzed using a bisulfite sequencing protocol in the FB, AB, D0, and D2 groups. The results are expressed as the means ± SD. “**” *p <* 0.01.

The results of a statistical analysis showed that the loci of Positions 6–8 in the AB group and in differentiated QCMCs had significantly higher DNA methylation levels in the FB group and inundifferentiated QCMCs (*p <* 0.01), respectively (Figure 2c and Figure 3). Thus, we hypothesized that the loci of Positions 6–8 in the core promoter (−216/−28) of the *SIX1* gene play major roles in regulating significantly higher DNA methylation levels.

### 2.3. Identification of the Histone H4 and E2F2 Binding Sites as Transcriptional Repressors in the Core Promoter Region of the SIX1 Gene by DNA Methylation

**Figure 4:**
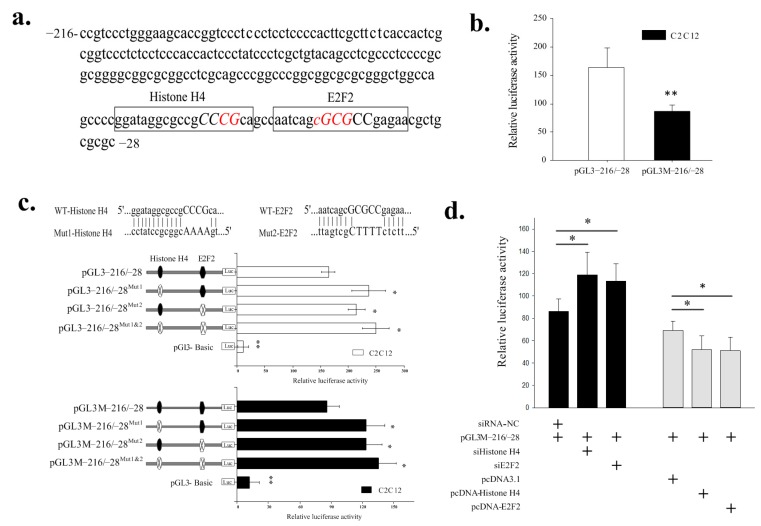
Functional analysis of the histone H4 and E2F2 binding sites as transcriptional repressors in the core promoter region of the *SIX1* gene by DNA methylation. (**a**) Sequence and putative transcription factor-binding sites in the core promoter of the *SIX1* gene. The putative transcription factor binding sites are boxed. The red sequences indicate methylation loci and capital letters indicate the core sequence of the transcription factors. (**b**) Analyses of *SIX1* promoter methylation through unmethylated and methylated luciferase reporter plasmids pGL3−216/−28. (**c**) Luciferase assays in constructs with site-directed mutagenesis of histone H4 and E2F2 binding sites were carried out with the construct pGL3−216/−28 and pGL3M−216/−28. Sequence graphical fill in the black and white are representative of wild and mutant-type, respectively. (**d**) Histone H4 and E2F2 knockdown and overexpression by the specific siRNA and pcDNA3.1 recombinant plasmids and co-transfected with pGL3M−216/−28 in C2C12 cells. The construction of pGL3−216/−28 and pGL3M−216/−28 denote the unmethylated and methylated luciferase reporter plasmids *in vitro*, respectively. The NC siRNA and pcDNA 3.1 (+) expression plasmid were used as a negative control. The results are expressed as the means ± SD in arbitrary units based on the firefly/Renilla luciferase activity. “*” *p <* 0.05 and “**” *p <* 0.01.

Analysis to identify regulatory elements in the *SIX1* promoter region was performed using the Matlnspector program (http://www.genomatrix.com) with a cutoff value over 90%. We identified the potential histone H4 and E2F2 TFs bind sites at Positions 6–8 in the −216/−28 region (Figure 4a). To investigate the roles of these sites in the methylation regulation of *SIX1*, we mutated the transcription factor-binding sites for histone H4 and E2F2 and generated the reporter constructs pGL3−216/−28, pGL3−216/−28^Mut1^ (mutation of histone H4), or pGL3−216/−28^Mut2^ (mutation of E2F2). These report plasmids were then methylated with methylase *M.Sss*I (New England Biolabs, Hitchin, UK) *in vitro*. The effects of unmethylated and methylated luciferase reporter plasmids (pGL3−216/−28, pGL3−216/−28^Mut1^, pGL3−216/−28^Mut2^, pGL3M−216/−28, pGL3M−216/−28^Mut1^, or pGL3M−216/−28^Mut2^) were transfected in C2C12 cells, which, based on our previous study, are superior cell models for determining the transcriptional activities of the *SIX1* gene. As shown in Figure 4b, the luciferase activity of pGL3−216/−28 was significantly higher than that of the pGL3M−216/−28 (*p <* 0.01). Mutation of the histone H4 or E2F2 sites in the construct pGL3−216/−28 resulted in a significant increase of 144% and 131%, respectively, in the *SIX1* promoter activity of C2C12 cells. Double mutations of the histone H4 and E2F2 binding sites resulted in an additional increase in the transcriptional activity of 152% compared with corresponding pGL3−216/−28 construction (Figure 4b). In addition, mutations of the histone H4 or E2F2 site in the construction pGL3M−216/−28 led to approximately 142–157% increases in *SIX1* promoter activity in C2C12 cells (Figure 4b).

Further validating the potential transcriptional repressors of histone H4 and E2F2, co-transfection of siRNAs against histone H4 and E2F2 into C2C12 cells dramatically increased pGL3M−216/−28 transcription levels (138% and 131%, respectively) (Figure 4c). Moreover, histone H4 and E2F2 overexpression significantly decreased the level of *SIX1* promoter activity of pGL3M−216/−28 in C2C12 cells (24.6% and 26.1% decrease, respectively). These results strongly suggest that the *SIX1* promoter activities are under the control of its promoter methylation. Furthermore, the histone H4 site (at Positions −75 to −58) and the E2F2 site (at Positions −54 to −38) were repressor binding sites and essential for basal transcriptional activity of the *SIX1* promoter by DNA methylation.

### 2.4. Histone H4 and E2F2 Bind to the SIX1 Promoter In Vitro and Vivo

**Figure 5:**
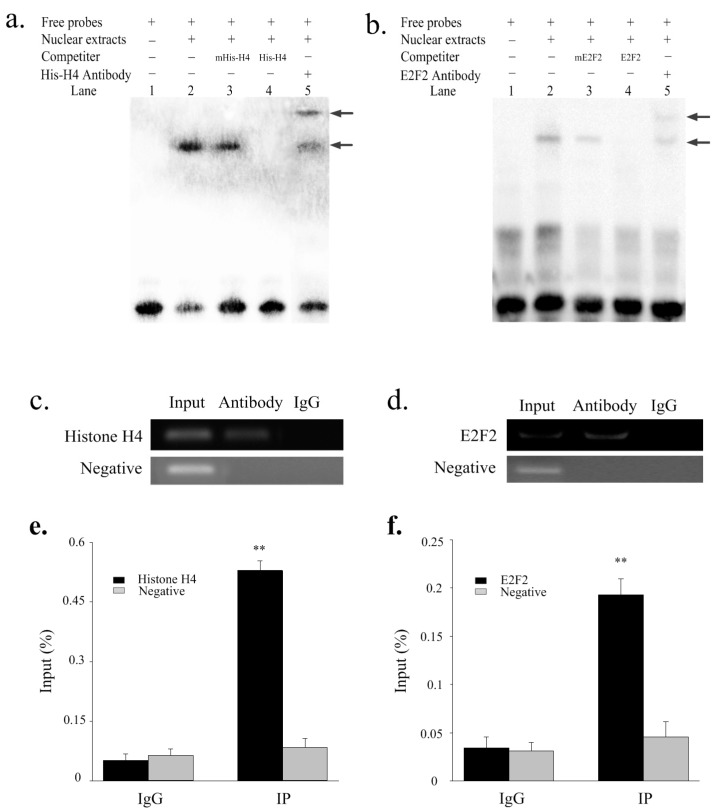
Electrophoretic mobility shift assay (EMSA) and ChIP analyses showing direct binding of histone H4 and E2F2 to the *SIX1* promoter *in vitro* and *in vivo*. (**a**,**b**) Lane 1: 5′-biotin labeled probe containing histone H4 and E2F2; Lane 2: histone H4 and E2F2 probes were incubated with nuclear extracts; Lane 3: the presence of histone H4 and E2F2 mutation probes (50×); Lane 4: histone H4 and E2F2 probes and nuclear extracts with 50-fold unlabeled oligonucleotides; Lane 5: histone H4 and E2F2 probes and nuclear extracts with 10 μg of anti-histone H4 or anti-E2F2 antibodies. The arrows mark the primary complex and super-shift brand. (**c**,**d**) ChIP-PCR products were analyzed with the input and immunoprecipitated products for histone H4 and E2F2. The enrichment of DNA fragments in samples immunoprecipitated with histone H4 (**e**) and E2F2 (**f**) antibodies via ChIP-qPCR. Normal rabbit IgG and an intragenic DNA fragment of *SIX1* exon 2 were used as negative controls. “**” *p <* 0.01. Error bars represent the SD (*n* = 3).

EMSAs and ChIP assays were used to confirm whether histone H4 and E2F2 bind to the *SIX1* promoter *in vitro* and *in vivo*. As shown in Figure 5a, the nuclear protein from undifferentiated QCMCs bound to the 5′-biotin labeled histone H4 probes and formed one primary band (Lane 2, Figure 5a). Competition assays showed that the mutant probe had little effect on the primary complex (Lane 3, Figure 5a). However, the primary band disappeared when the 5′-unlabeled histone H4 probe was added (Lane 4, Figure 5a). In addition, the complex was super-shifted when it was incubated with the histone H4-antibody (Lane 5, Figure 5a). The E2F2 results were similar to those observed for histone H4 (Figure 5b). The ChIP results revealed that histone H4 and E2F2 interacted with the binding sites (Figure 5c,d). The relative enrichment levels were ~8.8- and 4.6-fold over the IgG control, respectively (Figure 5e,f) based on three independent experiments.

## 3. Discussion

*The SIX1* gene has been highly conserved over evolution and plays a key role in the growth and development of diverse organisms, from the lower invertebrates to the higher vertebrates. In addition, the *SIX1* gene plays an important role in embryonic myogenesis and adult muscle regeneration in cooperation with MRF. A previous study showed that the *SIX1* gene is highly associated with DNA methylation during myoblast differentiation [15]. DNA methylation of CpG islands is one of the major epigenetic mechanisms controlling gene expression, with high CpG methylation inhibiting gene expression by decreasing promoter activity [16,17]. In our previous study, one CpG island (−216/−28) was predicted in the core promoter of the bovine *SIX1* gene. Therefore, we suspected that DNA methylation of the CpG sites in the *SIX1* gene promoter could alter the transcription activity and methylation regulation during development.

In the present study, the expression and DNA methylation levels of *SIX1* were determined in two key stages of myogenesis and muscle maturation. The relative gene expression levels of *SIX1* in muscle tissues in the FB group were significantly higher than in the AB group, which was similarly observed for the undifferentiated and differentiated stages of QCMCs. In addition, DNA methylation of bovine *SIX1* promoter significantly influenced the promoter activities and was negatively correlated with *SIX1* gene expression, which is consistent with previous findings observed in porcine *SIX1* methylation regulation [15]. DNA methylation is a post-replication modification that changes in an orchestrated way during cell differentiation and mammalian development [18,19,20]. Moreover, DNA methylation regulation mechanisms can respond to an organism adapting to the changes of environments through changes in gene expression [21,22]. In the present study, DNA methylation was observed to be higher in the AB and D2 group than in the FB and D0 groups, and we inferred that variations in DNA methylation patterns of the *SIX1* gene are sensitive to environmental factors after birth.

To further explore the methylation patterns of the *SIX1* gene, 9 CpG sites in the *SIX1* core promoter region that primarily contribute to the regulation of transcriptional activity were studiedin both the animal development group (AB and D2) and the myogenesis group (FB and D0). Three CpG sites (loci of Positions 6, 7, and 8) positively correlated with the DNA methylation levels in AB and D2 (*p* < 0.01). Analysis of a region of loci of Positions 6–8 in the bovine *SIX1* gene indicated potential binding sites for histone H4 and E2F2. Histone H4 belongs to the histone family and represents the most highly conserved chromatin protein in eukaryotes [23].Chromatin packaging of nascent DNA during S phase requires the cell cycle regulated expression of the histone H4 proteins [24,25]. In addition, the N-terminal tails of histone H4 play central roles in modulating nucleosome structure and functional processes by posttranslational modifications, including methylation, phosphorylation, and acetylation [26,27]. While histone acetylation and DNA methylation pathways can depend on each other [28]. In the present study, we observed that histone H4 mutation and knockdown increased the methylated transcriptional activity of the *SIX1* gene in C2C12. However, histone H4 overexpression led to a significant reduction in the basal activity of the unmethylated or methylated promoter region. The EMSA and ChIP results showed that histone H4 was capable of a high affinity to *SIX1*, suggesting that histone H4 contributes to regulation by methylation of *SIX1* in bovine skeletal muscle cells.

E2Fs are important regulators of proliferation, differentiation, and apoptosis [29]. To date, eight distinct genes (E2F1-8) encoding E2F proteins in mammals have been identified [30]. According to structural and functional analyses, the E2F family can be categorized into two general subclasses, transcription repressors (E2F1, E2F2, and E2F3a), and activators (E2F3b, E2F4, E2F5, E2F6, E2F7, and E2F8) [31]. Previous studies showed that E2F2-null mice were generally associated with a reduction in the proliferation capacity of cells [32], whereas an increase in E2F2 activity is often associated with inappropriate cell proliferation and/or apoptosis [33]. In addition, E2F2 is highly expressed specifically in oocytes and confers protection against de novo DNA methylation via nucleosome depletion by recruited Swi/Snf [33,34]. In support of the methylation regulation of gene transcription by E2F2, we observed that mutations and interference of the E2F2 greatly increased the activity of the unmethylated or methylated *SIX1* promoter, while E2F2 overexpression resulted in a reduced in promoter activity. EMSA and ChIP assays also demonstrated that these transcription factors could specifically bind sequences in the proximal promoter of *SIX1*.

## 4. Materials and Methods

### 4.1. Sample Collection

Samples of longissimus dorsi muscle were collected during five developmental stages from male Qinchuan cattle, including at 180 days in utero and at 1, 9, 18, and 24 months after birth. Three parallel animals were sampled during each stage. Both RNA and DNA were isolated within 10 min after slaughtering and at two key stages of myogenesis and muscle maturation samples, including 180-day-oldfetuses (fetal bovine, FB) and 24-month-old animals (adult bovine, AB) [35,36]. The animals used in this study were selected from farms and were reared in the same management and environmental conditions to reduce background error. Animals were fed the same feed of roughage at a concentrate ratio of 6:4 on a total mixed ration (TMR) basis. All animal procedures were performed according to guidelines laid down by the China Council on Animal Care (Ministry of Science and Technology of China, 2006), and the protocols were approved by the Experimental Animal Manage Committee (EAMC) of Northwest A&F University.

### 4.2. Quantitative PCR Analysis of Gene Expression Patterns

Longissimus thoracis were obtained from three parallel animals from the 180 days at the fetus stage and 1, 9, 18, and 24 months after birth. QCMCs were isolated from Qinchuan fetal bovine samples. For inducing QCMC differentiation, cells at 70% confluence were switched to differentiation medium containing DMEM/F-12, 2% horse serum (Gibco, Invitrogen, Carlsbad, CA, USA). Total RNA was extracted using RNAiso (Takara, Dalian, China) and reverse transcribed using a PrimeScriptTM RT Reagent Kit with gDNA Eraser (Takara) following the manufacturer’s instructions. An equal volume of cDNA was used for qRT-PCR. qRT-PCR was performed in triplicate using a SYBR^®^ Premix Ex TaqTM ΙΙ Kit (Takara) on an ABI 7500 Real-Time PCR system. Gene expression levels were normalized to that of *GAPDH*, and fold change was determined using the 2^−ΔΔ*C*t^ method [37]. The primers used for quantitative PCR analysis are listed in Table 1.

### 4.3. Western Blotting

For Western blot, tissue and cell proteins were extracted using T-PER Tissue Protein Extraction Reagent (Pierce, Thermo Fisher Scientific, San Jose, CA, USA). Protein was separated on 10% SDS-PAGE gels and incubated with the *SIX1* antibody (sc-514441, Santa Cruz, CA, USA) and GAPDH antibody (sc-293335, Santa Cruz, CA, USA). Signals were enhanced by ECL Plus (Thermo Scientific) and visualized by exposure of X-ray films to chemical luminescence using the ChemiDoc™ XRS+ System (Bio-Rad, Hercules, CA, USA).

### 4.4. DNA Preparation and Sodium Bisulfite Treatment and BSP

Genomic DNA was extracted with a TIANamp Genomic DNA Kit (Tiangen, Beijing, China) and prepared with sodium bisulfate using an EZ DNA Methylation Kit (Zymo Research, Irvine, CA, USA) according to the manufacturer’s protocol. Three separate bisulfite modification treatments were performed for each DNA sample. The BSP primers were designed by the online MethPrimer software [38] and are shown in Table 1. The PCR amplifications were performed in 20 μL volumes containing 50 ng of genomic DNA, 0.5 μM each of MSPF/MSPR primer (Table 1), and ZymoTaq Premix (ZymoTaq Premix, Zymo Research, USA) 10 μL. The PCR was performed using a program of 5 min at 95 °C, 38 cycles of 94 °C 30 s, annealing for 40 s at 50.4 °C and extension for 30 s at 72 °C. All PCR products were subcloned into the pMD19-T vector (Takara). Different positive clones for each subject were randomly selected for sequencing (Sangon, Shanghai, China). Finally, the sequences were analyzed using online QUMA software [39].

### 4.5. Potential TFs Luciferase Reporter Constructs and Substitution Mutation Constructs

The specific primers *SIX1*-P/R (−216/−28) was designed to contain the core promoter region of the bovine *SIX1* gene and ligated into the luciferase reporter construct pGL3-basic vector. The potential sites for histone H4 and E2F2 motifs were analyzed using the Genomatix suite (http://www.genomatix.de/) and mutated with the corresponding primers (Table 1) using a Fast Directed Mutagenesis Kit (Tiangen) according to the manufacturer’s protocol.

### 4.6. In Vitro Methylation

The series of recombinant luciferase reporter construct were methylated *in vitro* by using methylase *M.Sss*I (New England Biolabs) according to the manufacturer’s instructions. Briefly, the methylation reactions contained 2 μL diluted SAM buffer, 1 μg plasmid DNA, 4 U *M.Sss*I methylase, and 16 μL nuclease free water in a 20 μL total volume. Reactions were incubated at 37 °C for 2 h and purified with the QIAquick Nucleotide Removal Kit (QIAGEN, Valencia, CA, USA). The methylation state of promoter fragments was confirmed by digestion with methylation-sensitive restriction eyzyme *Nar*I (New England Biolabs). These methylated plasmids were named pGL3M−216/−28, pGL3M−216/−28^Mut1^, and pGL3M−216/−28^Mut2^.

### 4.7. Cell Transfection

C2C12 cells were cultured in DMEM containing 25 mM glucose supplemented with 10% newborn calf serum (NBCS; Invitrogen), 100 μg/mL streptomycin, and 100 U/mL penicillin at 37 °C and 5% CO_2_. Briefly, cells were grown in triplicate 24-well plates until reaching a density of 70%, after which 800 ng of the expression construct (pGL3−216/−28, pGL3−216/−28^Mut1^, pGL3−216/−28^Mut2^, pGL3−216/−28^Mut1&2^, pGL3M−216/−28, pGL3M−216/−28^Mut1^, pGL3M−216/−28^Mut2^, or pGL3−216/−28^Mut1&2^) was co-transfected with 10 ng of pRL-TK normalizing vector using Lip3000 (Invitrogen) according to the manufacturer’s instructions. The pGL3-basic vector served as a negative control. Forty-eight hours after transfection, the luciferase activities were measured using the Dual-luciferase^®^Reporter Assay System (Promega, Madison, WI, USA) the manufacturer’s instructions.The relative luciferase activities were determined using an infinite M200PRO NanoQuant Plate™ (TECAN, Hombrechtikon, Männedorf, Switzerland).

### 4.8. Histone H4 and E2F2 Knockdown and Overexpression

The interference efficiency of siRNA against histone H4 and E2F2 were screened and synthesized with the negative control siRNA (GenePharma Co., Ltd., Shanghai, China). The siRNA sequences are presented in Table 1. C2C12 cells cultured in 24-well plates were transiently co-transfected with 50 nM each siRNA with the corresponding pGL3M−216/−28. The pcDNA3.1-Histone H4 and pcDNA3.1-E2F2 expression plasmid was generated by reverse PCR to obtain the bovine histone H4 CDS and E2F2 CDS using specific primers (Table 1). For histone H4 and E2F2 overexpression, C2C12 cells cultured in 24-well plates were transiently co-transfected with 400 ng each of pcDNA3.1-Histone H4, pcDNA3.1-E2F2 and the corresponding pGL3M−216/−28. The pcDNA3.1 plasmid served as a negative control. 

### 4.9. Electrophoretic Mobility Shift Assays (EMSAs)

Nuclear extracts were obtained from undifferentiated QCMCs according to the manufacturer’s protocol and used for EMSAs using LightShift Chemiluminescent EMSA Kit (Thermo Fisher Corp., Waltham, MA, USA) with modifications. Briefly, 200 fmol of 5′-biotin labeled histone H4 or E2F2 probes (listed in Table 1) was incubated with 10 μg nuclear extracts, 2 μL of 10 × binding buffer, 1 μL of poly dI·dC and 1 μL of 50% glycerol in a volume of 20 μL. For the competition assay, unlabeled or mutated probes were added to the reaction mixture for 10 min before adding the labeled histone H4 or E2F2 probes. Finally, 10 μg of histone H4 (ab5823, Abcam, Cambridge, MA, USA) or E2F2 (ab138515, Abcam) antibody was added to the reaction mixture for the super-shift assay. The DNA-protein complexes were separated on a 6% non-denaturing polyacrylamide gel and based on three independent experiments. Images were captured using the molecular imager ChemiDoc™ XRS+ system (Bio-Rad, Hercules, CA, USA).

### 4.10. Chromatin Immunoprecipitation (ChIP) Assay

Longissimus thoracis samples were obtained from three parallel Qinchuan cattle at 3 days after birth (*n* = 3) and used for ChIP assays by with the Simple ChIP^®^ Enzymatic Chromatin IP kit (CST, Danvers, MA, USA) according to the manufacturer’s instructions. The protein-DNA complexes were cross-linked and immunoprecipitated with 4 μg of histone H4 or E2F2 antibodies overnight at 4 °C, and the immunoprecipitated products were collected with Protein A+G coated magnetic beads. Next, we purified the DNA for PCR analysis. The ChIP-Histone/E2F2 was used in standard PCR and quantitative real-time PCR experiment (Table 1). ChIP-PCR amplification was verified by electrophoresis of the products in a 2% (*w*/*v*) agarose gel. ChIP-qPCR was calculated as follows: % Input = 2^[−Δ*C*t(*C*t[ChIP]−(*C*t[Input]−Log2(Input Dilution Factor)))]^ [40]. We used normal rabbit IgG and an intragenic DNA fragment of the *SIX1* exon 2 (ChIP-Control) as negative controls.

### 4.11. Statistical Analysis

Data are expressed as mean ± standard deviation (mean ± SD) from three independent experiments. The levels of gene expression, DNA methylation, and luciferase assays based on three independent experiments and unpaired Student’s *t*-test were used to detect significant differences. “*” *p <* 0.05 and “**” *p <* 0.01.

## 5. Conclusions

**Figure 6:**
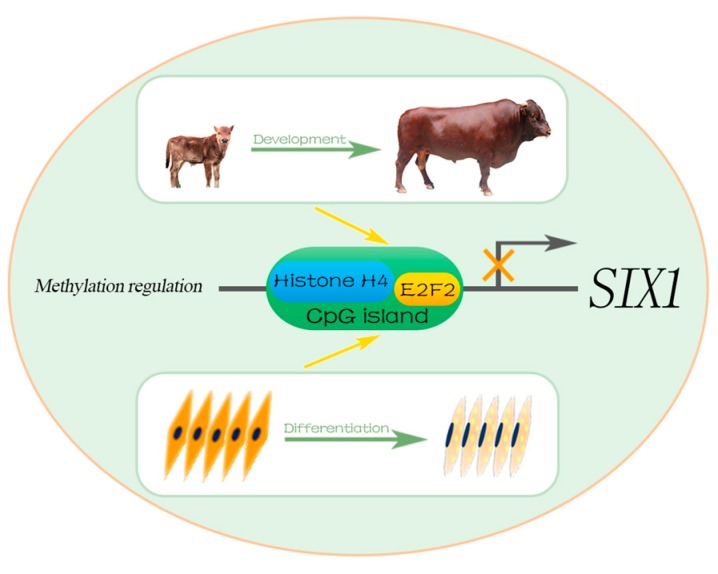
A hypothesized methylation regulatory mechanism of bovine *SIX1*. The light green circle shows a specific methylation regulation progress of the bovine *SIX1* promoter was regulated by histone H4 and E2F2 during the development. The green arrows indicate the progress of individual development and muscle cells differentiation of Qinchuan cattle. The yellow arrows indicate repression of *SIX1* promoter activity by DNA methylation through histone H4 and E2F2 TF during development.

In summary, the expression of bovine *SIX1* was significantly higher in the FB and in undifferentiated QCMCs. Three CpG sites in the core promoter region of the bovine *SIX1* gene were identified in the contribution significantly higher DNA methylation levels. In addition, we found that the DNA methylation of *SIX1* promoter *in vitro* significantly influences the promoter activities and the DNA methylation level of *SIX1* promoter core region was negatively correlated to its expression level. Furthermore, Histone H4 and E2F2 binding occurs in the three CpG sites and plays important roles in bovine *SIX1* methylation regulation. Thus, a hypothesized regulation methylation mechanism of *SIX1* is shown in Figure 6. The results of this study provide foundational information for understanding the methylation regulation and biological function of the *SIX1* gene during bovine myogenesis.

## Figures and Tables

**Table 1:** Primers used in the expression and methylation analysis of the *SIX1* promoter experiments.

Reaction	Name	Primer Sequence (5′ to 3′)	Temperature (°C)	Product Length (bp)	Amplified Region
qRT-PCR	GAPDH	F: CCAACGTGTCTGTTGTGGAT R: CTGCTTCACCACCTTCTTGA	60.0	80	778/857
	*SIX1*	F: GCCAAGGAAAGGGAGAACA R: GACTCTGGGGAGGTGAGAACT	60.0	127	866–992
Promoter cloning	*SIX1*-F/R	F: **CGGGGTACC**CTCACGTTGCAAGGTCCTGAC R: **GGAAGATCT**GCGTTCTCGGCGCGCTGATTG	61.5	189	−216/−28
Methylation analysis	*SIX1*-MSF/MSR	F: TCGTTTTTGGGAAGTATTGGT R: ACACACAACGTTCTCAACG F: TTGTTTTTGGGAAGTATTGGT R: ACACACAACATTCTCAACA	50.4	189	−216/−28
	*SIX1*-USF/USR		51.5	189	−216/−28
Site-mut and EMSA	Histone H4 forward	CCCGGATAGGCGCCG CCCGCAGCCAATCAGCGCG			−51/−17
	Histone H4 reverse	CGCGCTGATTGGCTGC GGGCGGCGCCTATCCGGG			
	mHistone H4 forward	CCCGGATAGGCGCCG AAAACAGCCAATCAGCGCG			−51/−17
	mHistone H4 reverse	CGCGCTGATTGGCTGC TTTTGGCGCCTATCCGGG			
	E2F2 forward	CCGCAGCCAATCAGC GCGCCGAGAACGCTGCGCG			−35/−2
	E2F2 reverse	CGCGCAGCGTTCTC GGCGCGCTGATTGGCTGCGG			
	mE2F2 forward	CCGCAGCCAATCAGC GAAAAGAGAACGCTGCGCG			−35/−2
	mE2F2 reverse	CGCGCAGCGTTCTC TTTTCGCTGATTGGCTGCGG			
ChIP	ChIP-Histone H4/E2F2	F: CCACCACTCCCTATCCCTCGCTGTA	60	196	−145/50
		R: TGCCGGGGAACTTGGTTTCTGTT			
	ChIP-control	F: GTTTTGTTTACCACTAGCTTTTC	60	134	Exon 2
		R: ATCCTTGTAGGAGTTCCCTTT			
siRNA	siHistone H4	UGUGACUGUUGUGACUACAGCUGUATT			
	siE2F2	GAUAUCCGUGCCGUAGGCAACUUCATT			
	siRNA-NC	UUCUCCGAACGUGUCACGUTT			
Overexpression	Histone H4-CDSF/R	F: **CCGATATC**ATGCCGCCACCTGGGAAAGTTC	62	1551	NM_001038531
		R: **GCTCTAGA**TCACACCATCTGGACTTCTGG			
	E2F2-CDSF/R	F: **CCGATATC**ATGTGGGGTAGGCCCCACCCA	63.5	1299	XM_869196
		R: **GCTCTAGA**TCAGTTAATCAGCAGGTCCC			

Forward (F) and reverse (R) primers promoter cloning and overexpression were designed with *Kpn*I, *Bgl*II, *EcoR*V, and *Xba*I restriction sites (bold letters), respectively. The oligonucleotides and probes used forEMSA contained putative core transcription factor-binding sites (underlined).

## Abbreviations

TF	transcription factor
FB	fetal bovine
AB	adult bovine
QCMCs	Qinchuan cattle muscle cells
MRFs	myogenic regulatory factors
BSP	bisulfite sequencing polymerase
EMSA	electrophoretic mobility shift assay
ChIP	chromatin immunoprecipitation
TSS	transcriptional start sites
MSP	methylated-specific primer
USP	unmethylated-specific primer
